# SPTV sheds light on flow dynamics of fractal-induced turbulence over a plate-fin array forced convection

**DOI:** 10.1038/s41598-021-02872-1

**Published:** 2022-01-07

**Authors:** Su Min Hoi, Ean Hin Ooi, Irene Mei Leng Chew, Ji Jinn Foo

**Affiliations:** School of Engineering, Monash University Malaysia, 47500 Bandar Sunway, Selangor Malaysia

**Keywords:** Fluid dynamics, Techniques and instrumentation, Physics, Engineering, Mechanical engineering

## Abstract

A 3D stationary particle tracking velocimetry (SPTV) with a unique recursive corrective algorithm has been successfully established to detect the instantaneous regional fluid flow characteristics. The veracity of SPTV is corroborated by conducting actual displacement measurement validation, which gives a maximum percentage deviation of about 0.8%. This supports the accuracy of the current SPTV system in 3D position detection. More importantly, the SPTV detected velocity fluctuations are highly repeatable. In this study, SPTV is proven to be able to express the nature of chaotic fractal grid-induced regional turbulence, namely: the high turbulence intensity attributed to multilength-scale wake interactions, the Kolmogorov’s −5/3 law decay, vortex shedding, and the Gaussian flow undulations immediately leeward of the grid followed by non-Gaussian behaviour further downstream. Moreover, by comparing the flow fields between control no-grid and fractal grid-generated turbulence of a plate-fin array, SPTV reveals vigorous turbulence intensity, smaller regional integral-length-scale, and energetic vortex shedding at higher frequency for the latter, particularly between fins. Thereupon, it allows the unravelling of detailed thermofluid interplays of plate-fin heat sink heat transfer augmentation. The novelty of SPTV lies in its simplicity, use of low-cost off-the-shelf components, and most remarkably, low computational complexity in detecting fundamental characteristics of turbulent fluid flow.

## Introduction

Fluid transportations are fundamentally crucial in numerous scientific and technological problems, including but not limited to biophysics^1,2^, process engineering^3,4^ and thermal management^5,6^. The most common type of fluid flow found in nature and has been predominantly exploited in a variety of engineering applications is turbulence^7,8^. Such flow dynamic is highly chaotic yet diffusive, which contributes significantly to momentum, heat and mass transfer in flows of practical interest. More importantly, the capability of turbulent motion to facilitate the continuous reshuffling of flow and thermal boundary layers is vital to potently enhance forced convective thermal dissipation^9–12^. Such desirable flow characteristics have drawn much attention in deciphering the chaotic nature of turbulence^13–19^. Multiple turbulence generators continue to be invented and explored to effectively produce specific fluid flow perturbation. To date, the most well-known passive turbulators are the 2D planar space-filling biplane square grid and fractal insert. Fractal is a geometrical structure having self-similarity shapes at all scales that form an intricate pattern of different number of iterations^20^. Recently, it has become the focus of multitudinous investigations owing to its exciting performance in producing multilength-scales turbulence, paving the way to tune fluid flow dynamically. Interestingly, the turbulent flow generated using 2D planar space-filling fractal grids embodies more intermittent or more clustered vorticity field and higher turbulence intensities as compared to fluid flow undulations induced via the conventional regular grids of equal or higher blockage ratio^21–24^.

The understanding of turbulence is indispensable for the development or optimization of technological applications and industrial processes. Heating, Ventilation and Air Conditioning (HVAC) systems have been the subject of intensive studies in recent years^25–28^. HVAC systems provide good indoor air quality and desirable thermal environments, yet it consumes about 40% of the total energy in a commercial building^29^. The manifestation of the foremost heat exchanging element in HVAC systems, the finned-tube heat exchanger, contributes significantly to energy consumption. An enormous number of engineering design and research are being invested in promoting the efficiency of heat exchangers by intensifying the air-side heat transfer coefficient. One of the renowned methods is to introduce effective fluid flow perturbations across finned-tube heat exchanger^30–34^. The turbulent fluid flow fluctuations allow the restructuring of flow-boundary layers within the highly compacted finned-tube heat exchangers to promote forced convection. Cafiero et al.^35,36^ experimentally reported that fractal grid is able to significantly augment circular impinging jets forced convection as compared to a regular grid of equivalent blockage ratio and pumping power due to the high turbulence intensity and high magnitude and persistence streamwise vorticity immediate leeward of the fractal grid. Furthermore, several investigations have revealed the fact that vortex shedding, as well as turbulence integral length scale, play critical roles in thermal dissipation^12,37,38^. Although many studies have unravelled the undertaking of turbulence integral length scale upon forced convective heat transfer, the basic correlation of both remains contentious^12,37–41^.

Reliable characterization of fluid flow phenomena is the key to effectively disclose the first principle understanding of turbulence. In the past decade, dozens of flow measurement and visualization techniques have been devised to quantitatively detect turbulence. The well-known instrumentations are hot-wire anemometry (HWA), particle image velocimetry (PIV), and particle tracking velocimetry (PTV). HWA, which has high accuracy and sensitivity, is one of the main techniques that has been extensively employed in the studies of fractal-induced turbulence^21,42–44^. It is a point-wise local flow velocity measurement that extracts only the flow dynamics proximate to the sensor probe. However, such approach is invasive, which may unavoidably disturb the near-probe flow field. Thus, HWA is rather inappropriate for large gradient fluid flow detection, e.g., along the flow boundary layers, as well as the expression of regionally correlated vortices^45^. Besides, the application of HWA is strictly probe dimensions dependent, viz., it makes the flow dynamic measurement within a highly compact HVAC finned-tube heat exchangers of about 1.5 to 2.0 mm inter-fin spacing impossible. Alternatively, tracer-based optical velocimetry, e.g., PIV and PTV, enables non-intrusive flow velocity detection in a global domain, which allows the realization of flow velocity through the reconstruction of optically sensed tracer particles’ displacements in flow^26,46–48^.

PIV is considered the most renowned optical velocimetry that is specifically designed for airflow measurements. Numerous developments have been conducted since its first appearance in Adrian^49^ in the 1980s^47,50–55^. It provides two-dimensional simultaneous velocity vectors of multiple tracer particles by averaging the motion of particles in subsections. This velocity computation approach smooths the velocity field within the interrogation areas causing PIV to be impractical for measuring flow dynamics with large velocity gradients^56,57^. On the other hand, PTV is a Lagrangian oriented concurrent tracer particle tracking, which follows and tracks individual particle^26^. Thus, it is not surprising to perceive the increasingly reinvigorated interest in the applications of PTV to secure fluid flow observation with higher temporal and spatial resolutions, and more importantly, longer trajectories^26,58–62^. The downside of PTV, however, is that it requires a tremendously complex tracking system to thoroughly follow the motion of all seeding particles^56^. More importantly, a significant number of computer processors are required to effectively support the parallelized tracking algorithm^63–66^. Thus, a balance between HWA, PIV and PTV is of great importance in understanding the efficacy of fractal grid-induced turbulence on passive heat sink forced convection. In this study, a practical, non-intrusive and low-computational cost in-house stationary-particle tracking velocimetry (SPTV) is carefully designed, set up, validated and developed with the key objective of unveiling the fundamental correlation between fractal-generated turbulence and plate-fin array forced convective heat transfer augmentation. Evidently, in-depth comprehension of fractal-induced turbulence and its implications on thermal enhancement may shed light on the research and development of efficient heat exchangers that can pave the way towards long-term energy sustainability.

## SPTV system

**Figure 1:**
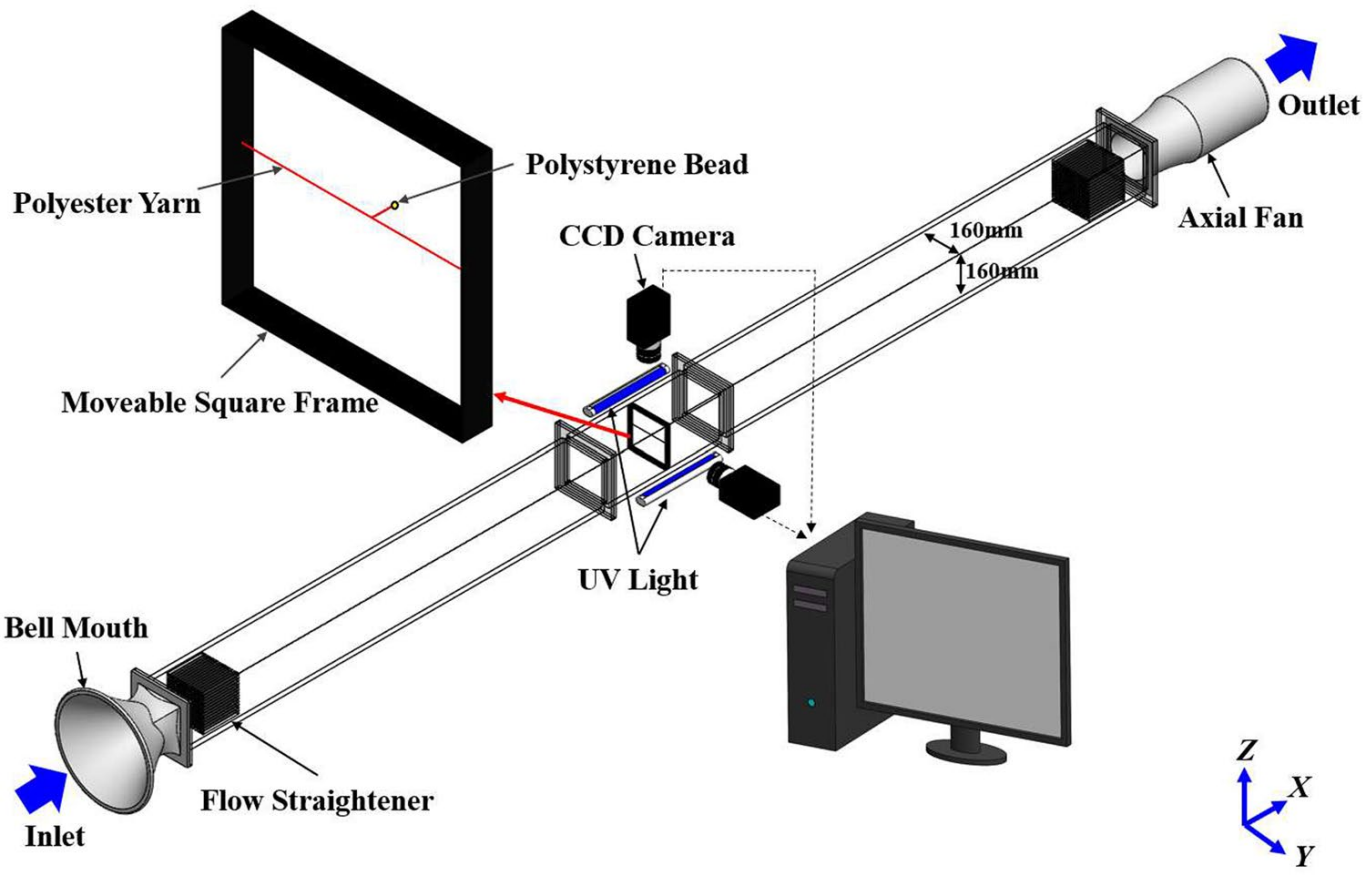
Schematic of the present SPTV experimental setup.

**Figure 2:**
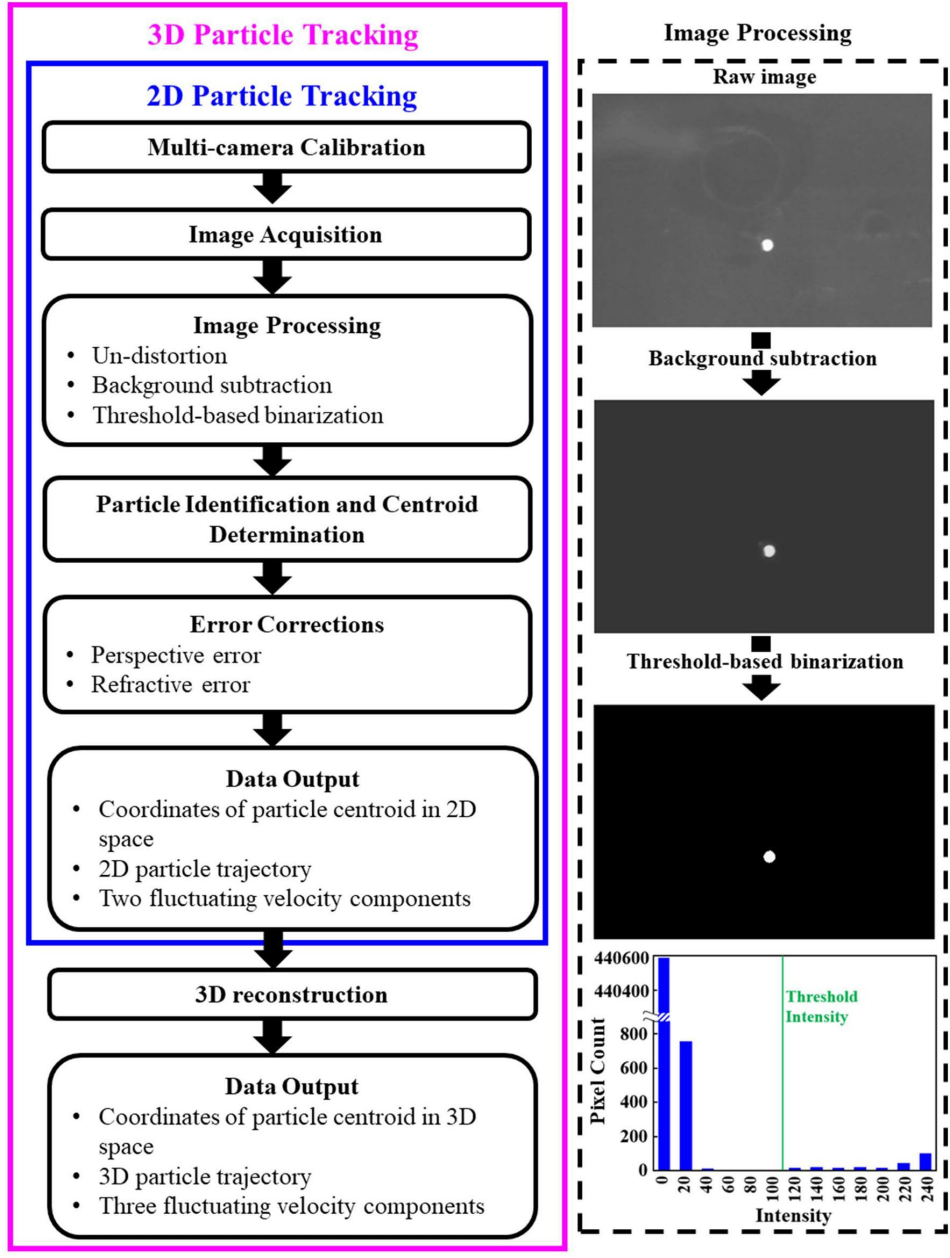
Left: Flow chart of SPTV velocimetry. Right: Demonstration of image processing method and determination of threshold intensity for threshold-based binarization.

**Figure 3:**
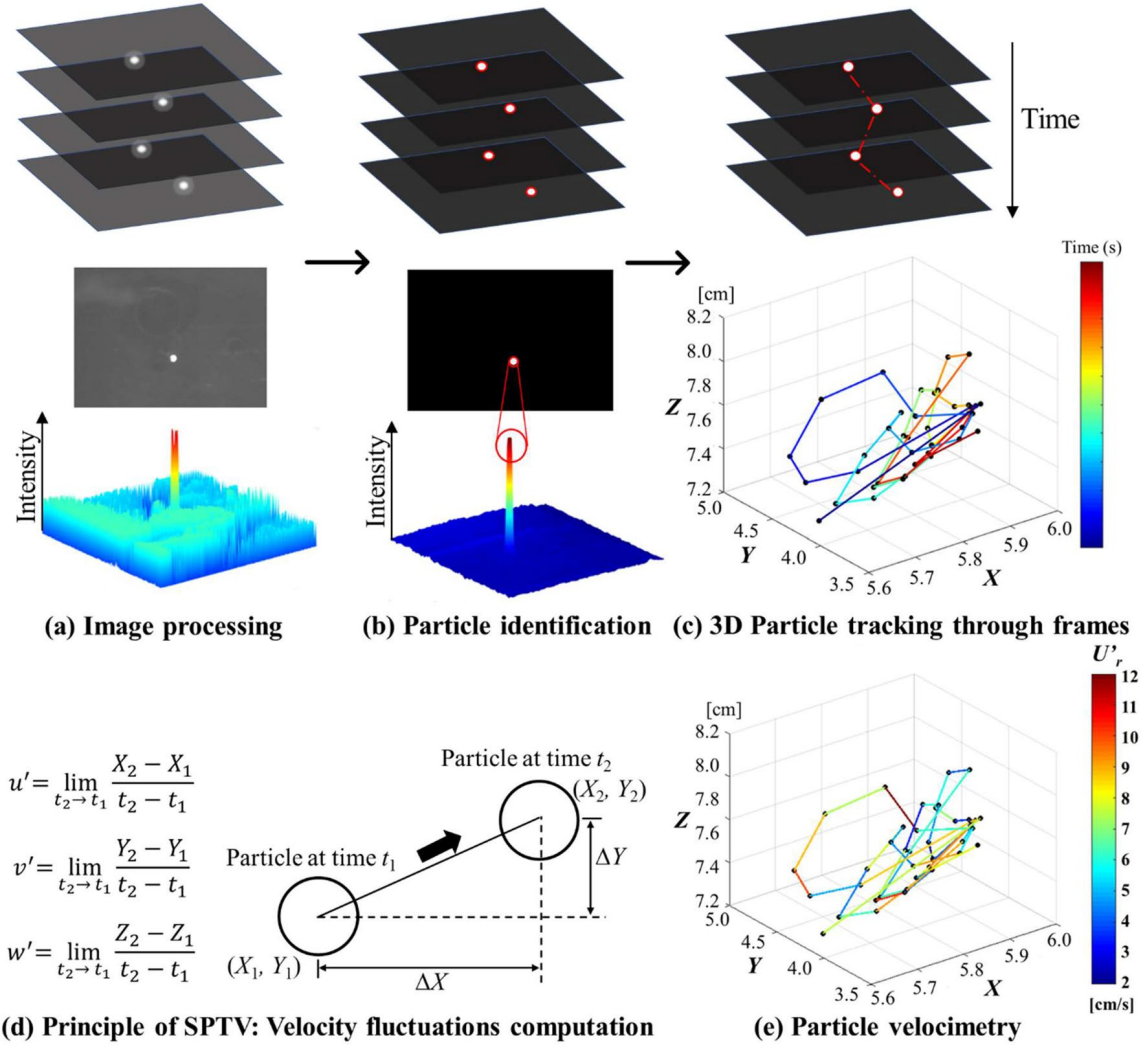
Overview of SPTV workflow: (**a**) image processing including image un-distortion, background subtraction and threshold-based binarization, (**b**) particle identification and centroid determination, (**c**) 3D reconstruction and particle trajectory (image frame 1 to image frame 40), (**d**) the fundamental principle of SPTV: velocity fluctuations computation, and (**e**) 3D particle velocimetry (image frame 1 to image frame 40).

The present SPTV, like PTV, tracks particle in consecutive image frames and computes the velocity from the displacement of particle within a prescribed time interval. SPTV comprises four major components, namely: (i) the illumination system, (ii) tracer particle, (iii) image recording devices, and lastly (iv) the post-image processing and evaluation algorithms [see Fig. 1]. Interestingly, unlike the typical PTV systems, where multiple unrestrained particles are used, SPTV employs only one tracer particle that is connected to a thin polyester yarn, which the yarn is then attached to a square frame (Fig. 1). The tethering of tracer particle to the square frame via polyester yarn allows the particle to fluctuate only within a “local” region in a fluid flow, depending on the length of the string it is suspended to, instead of following the working fluid to scatter further downstream of a wind tunnel. Although tethering or localizing the particle precludes translation in the downstream direction, it extends the observation time, which allows the particle to adequately explore the entire local volume, providing more detailed information in a particular region than the conventional ensemble-averaged method. In other words, SPTV can retrieve velocity fluctuations, specifically, the precious insert-induced fluid flow perturbation statistics within one particular region of interest, for e.g., the confined workspace between fins. A detailed workflow of the three-dimensional SPTV is outlined in Figs. 2 and 3.

### Illumination system

Two 8 W ultraviolet (UV) lights (Philips, JP) are employed to produce uniform, continuous and steady illuminations onto a fluorescent tracer particle that is partitioned within a dark observation volume. Such apparent contrast between the illuminated particle and concealed dim imaging space facilitates the generation of high signal to noise ratio time-series particle images. The merits of the current UVs setup ensure high luminous efficiency together with low heat dissipation, which reduces the influence of thermally driven hydrodynamics.

### Tracer particle

The identification of an appropriate tracer particle such that it faithfully follows the fluid element without disturbing the flow requires thorough consideration. Various important features determine the tracking performances of a tracer particle, i.e., shape, surface roughness, size and density. An ideal tracer particle has spherical geometry, smooth surface, neutrally buoyant, good light scattering efficiency, long lifetime, and non-evaporative^26,54,58,60,66^. However, attaining an optimal flow marker is a real challenge, especially for airflow due to the low air density. A tracer particle should not only be small enough to warrant high flow tracking accuracy but also allow the scattering of adequate signals to imaging devices under an appropriate level of illumination^47,60^. Hence, a compromise between particle size and light scattering efficiency is essential. For the current SPTV development, a yellow fluorescent spherical expanded polystyrene (EPS) particle of diameter *d*_*p*_ = 1 mm, density *ρ*_*p*_≈21.5 kg/m^3^ and refractive index of 1.58 (which permits preferable light scattering characteristic) is employed. The particle’s high value of luminosity and homogeneous light scattering intensity allow the realization of consistent and eminent signal-to-noise ratio upon time-series SPTV imaging. Lastly and most importantly, the fluorescent particle is attached to a thin polyester yarn (diameter≈2 × 10^-4^ m, length = 2 × 10^-2^ m), which is then incorporated to a transportable frame. This setup allows the bead to fluctuate within any specific region of interest along a wind tunnel [see Fig. 1].

The Stokes number *St* is widely recognized as an indicator for particles traceability. *St* is defined as the ratio of the particle response time *τ*_*p*_ to the characteristic timescale of the fluid flow *τ*_*f*_ :

1 $$St = \frac{{\tau_{p}^{1} }}{{\tau_{f} }}$$

where the particle response time *τ*_*p*_ is

2 $$\tau_{p}^{1} = \frac{1}{18}\left( {\frac{{\rho_{p}^{1} }}{{\rho_{f} }}} \right)\frac{{d_{p}^{2} }}{\nu }.$$

As high energy-containing range is the scale of interest in this study, the characteristic flow time scale *τ*_*f*_ is the integral time scale expressed as *T*_*L*_ = *L*_*i*_*/u’*, where *L*_*i*_ is integral length scale and *u’* is horizontal velocity fluctuation^67,68^. Using this definition for the characteristic flow time scale, the *St* ranges from *St* = 1.4 × 10^−2^ to 5.5 × 10^–2^, which is smaller than the recommended *St* of 0.1^26,54,69^. This suggests that the tracer particle characterizes the large scale flow patterns appropriately.

### Image acquisition system

#### Image recording devices

Coupled charged device (CCD) camera has been widely used in PTV experiments^46,53,58,59,61,70,71^, as it offers excellent quality images, high light sensitivity, high signal to noise ratio, and convenient data transmission^26,53^. Hence, a pair of high-speed CCD cameras (FLIR Integrated Imaging Solutions Inc., CA) equipped with wide-angle lenses (Tamron 25 mm F/1.4–22, US) are integrated into the SPTV system to experimentally record and decipher the details of regional flow field induced tracer particle’s undulated trajectories. Each monochrome camera has a resolution of 1920 × 1200 pixels (pixel size of 5.86 μm), frame rate of 80 fps, 1.112 ms exposure time and 8 bits per pixel sensor rate. Both cameras are respectively mounted onto the camera position alignment controllers (JDM Technologies, MY) placed perpendicular to each other at the top and side of the test section. The cameras are connected to a PC (USB 3.0) and controlled using FlyCapture-2 data acquisition platform.

#### Multi-camera calibration

**Figure 4:**
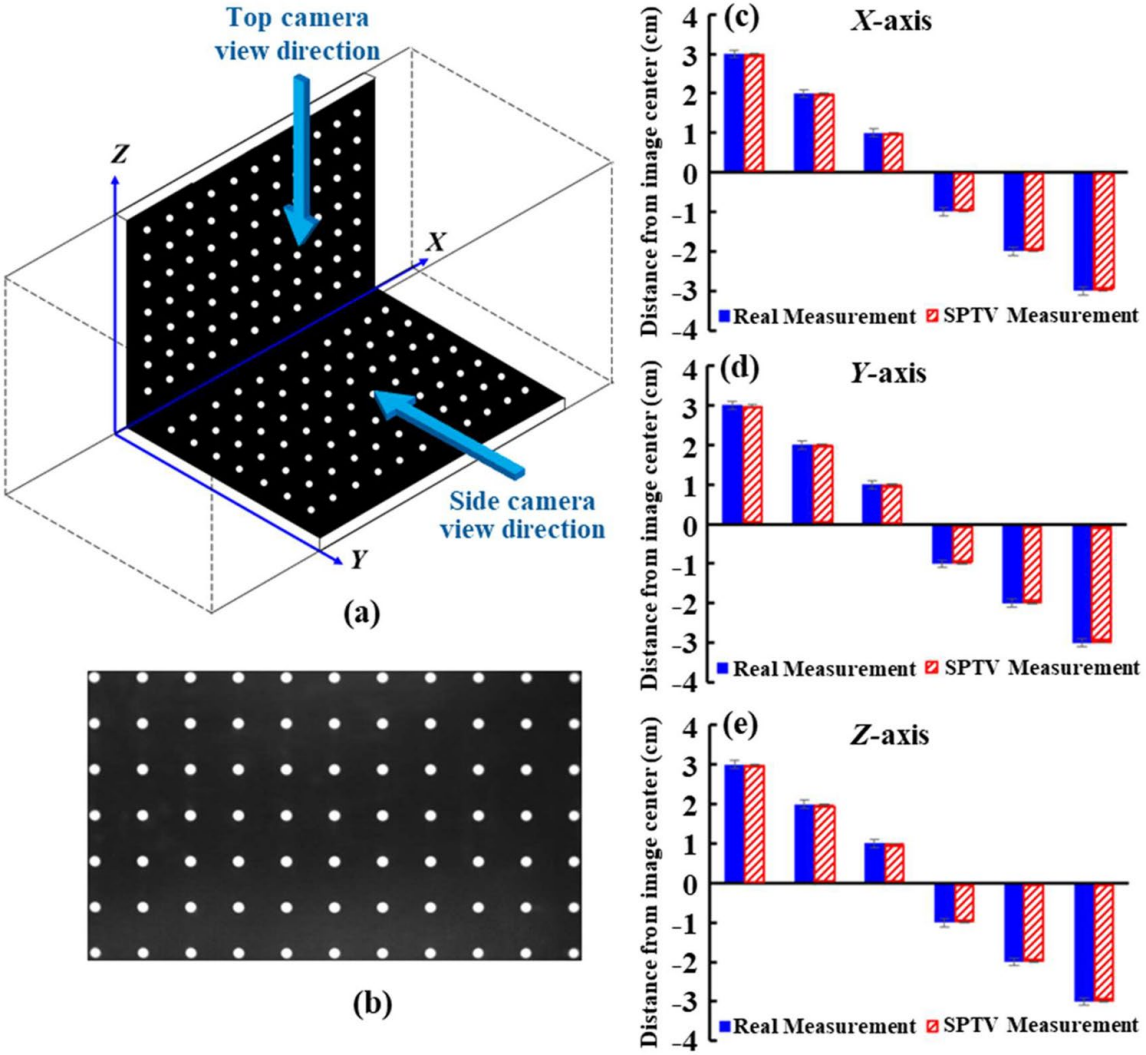
System calibration: (**a**) *L*-shape position calibration platform, (**b**) the camera-view, as well as the calibrated SPTV precision along the (**c**) *X*-, (**d**) *Y*-, and (**e**) *Z*-axis with respect to control positions.

It is indispensable to deduce the experimental correlation and the incorporation of each camera’s 2D pixel coordinates into the 3D Cartesian coordinate system via multi-camera calibration. Briefly, an *L*-shaped calibration platform that consists of two flat boards, assigned along the *X*–*Y* and *X*–*Z* planes of the test section and filled with an 11 × 7 array of equally spaced white circles, is employed to realize a precise alignment between the optical axis of each camera [see Fig. 4a]. Both cameras are adjusted perpendicularly with respect to each other by referring to the calibration platform, based on the individual frontal view as given in Fig. 4b. An in-house algorithm developed using MATLAB (R2016b, US) is applied to determine the position of the centre circle on the calibration platform and its deviation from the camera optical axis. Subsequently, both cameras’ orthogonal axes are aligned strictly perpendicular to each other by offsetting the deviations using camera position alignment controllers (streamwise and spanwise sensitivities: 0.10 mm and 0.01 mm, respectively). Hence, a well-calibrated multi-camera SPTV system simplifies the algorithm for the later 3D fluid flow reconstruction.

#### Multi-camera synchronization

Multi-camera synchronization is crucial in accomplishing the accurate time-dependent 3D reconstruction of flow marker’s trajectories. An external trigger is installed and employed to activate and synchronize the simultaneous imaging processes. The synchronization is validated by introducing a flashlight illumination such that a sudden increase in background intensity appears at the predefined frame.

### Image processing

Several image denoising approaches are applied [see Fig. 2] to enhance the contrast between the illuminated tracer particle and the corresponding background, such that the tracer particle’s motion can be detected accurately using custom code in MATLAB.

#### Lens distortion calibration (image un-distortion)

Lens distortion that results in image deviation can be compensated by identifying the camera’s intrinsic and extrinsic parameters via camera self-calibration. Briefly, images of a 2D planar checkerboard with a 5 × 8 array of 9.5 mm squares positioned at 25 orientations via any combination of its: (i) rotations about the optical axis, (ii) rotations less than 45 degrees relative to the camera plane and (iii) translations across the image plane, without changing its distance from the camera, are captured. The calibration parameters, e.g., focal length, distortion coefficients, and rotation/translation matrices, are acquired from the 25 images using MATLAB single-camera calibration toolbox. Based on the distortion coefficients determined from the camera calibrator, all the time-series particle images are undistorted using MATLAB built-in function *undistortImage* algorithm.

#### Background subtraction

MATLAB stores 8 bits grayscale images in pixels, and each pixel has a value ranging from 0 to 255, which describes its intensity brightness. An image of the background (flow field without tracer particle) is captured for each camera. Subsequently, the pixel values of the background image are subtracted from every recorded image to reduce the background noise [see Fig. 2].

#### Threshold-based binarization

To further enhance the contrast between the particle and its surroundings, an image segmentation method—fixed-level binary thresholding is employed to convert intensity images into binary images containing only 0 s and 1 s, which represent black and white, respectively. A threshold intensity of 110 is selected based on a gray level histogram for the current SPTV system [see Fig. 2]. Any pixels with a pixel value higher than the threshold intensity are set to one, whereas set to zero otherwise. After the image binarization, the non-uniformities of background intensity level due to reflections are removed, and the high signal-to-noise ratio images are then adequate for particle detection [see Figs. 2 and 3].

### Particle identification and centroid determination

For circular particle identification and centroid determination, circular Hough transform (CHT) is applied to extract circular particles from SPTV recorded images. In CHT, each pixel defining the boundary of the circular particle (identified via gradient-magnitude edge detection) on image space contributes to a circle in parameter space where every single point forming the circle represents a vote. The votes are accumulated in an accumulator array, and the coordinate with the highest number of votes (greatest number of circles passing through) corresponds to the centroid of the particle centre^72,73^.

### Error correction

Potential errors must be resolved to prevent degradation of image quality, which potentially contribute to errors in particle coordinate identification. Refraction, which occurs when light is deflected in passing obliquely through a medium of varying density, causes the tracer particle to appear at an offset distance from the actual position. A simple module advocated by Maas et al.^58^ is employed to incorporate refraction correction into the present SPTV algorithm. The module utilizes Snell’s Law to compute the radial deviation of each object point with respect to the camera optical path. Perspective error, too, exists in the imaging system, in which the magnification of an object depends on its distance from the lens. The impact of the perspective effect is significant, especially in measuring objects with depth or objects moving relative to the camera lens. The perspective effect is eliminated by solving perspective projection equations derived through a similar triangles rule associated with the particle coordinates on the image plane and optical centre plane.

### Particle tracking

The particle tracking strategy employed in SPTV involves particle tracking in the 2D image domain of each camera independently through time-based over the successive frames, followed by 3D reconstruction via spatial matching of the 2D trajectories [see Fig. 2]. The 3D reconstruction algorithm is simple and highly effective as the images are taken with strictly calibrated cameras. The first camera in the perpendicularly aligned camera system delivers the particle centroid coordinates in the *X*–*Y* plane, while the second camera delivers coordinates in the *X*–*Z* plane. The reconstruction step combines 2D coordinates of particle centroids from the *X*–*Z* and *X*–*Y *planes, such that one integrated set of 3D coordinates in (*X*, *Y*, *Z*) and the corresponding particle trajectory can be realized [see Fig. 3c]. This visualization provides a general idea of how a particle moves within the region of interest. Next, the instantaneous velocity fluctuations of the tracer particle in all directions are calculated by computing the changes in displacement over the time interval between frames [see Fig. 3d, e].

### Assessment of tracer particle position accuracy

One of the main concerns for the current SPTV is its ability to attain accurate tracer particle position detection, which is the primary information for subsequent data computations and evaluations. Thus, SPTV displacement validation is performed to ascertain the precision of camera alignments, as well as the accuracy and reliability of the SPTV algorithms. Images of a 1 mm fluorescent particle located at 1, 2 and 3 cm from the centre of the image frame in *X*, *Y* and *Z* directions, which guarantee complete coverage of the field of view, are taken. Later, the imaged particle displacements from the centre of the image frame are computed via the SPTV algorithm. Figure 4(c-e) compares the error margin between the assigned real displacement and the corresponding SPTV measurements along *X*, *Y* and *Z* directions. Evidently, the SPTV measurements match reasonably well with the actual distance detected. The maximum percentage differences measured are 0.43%, 0.51% and 0.78% for (*X*, *Y*, *Z)*, which correspond to the absolute value of 4.3 × 10^−3^ cm, 5.1 × 10^−3^ cm and 7.8 × 10^−3^ cm, respectively. Such validation assures the current SPTV’s ability to acquire data with high positional accuracy and reliability.

## Experimental setup for SPTV application

### Wind tunnel

The SPTV system is used to unravel the fractal-generated turbulent flow fluctuations before, within and after the transparent plate-fins in a wind tunnel of *L*_*x*_ × *L*_*y*_ × *L*_*z*_ = 4500 × 160 × 160 mm^3^. The wind tunnel is made of acrylic to facilitate optical access for the SPTV technique. It is equipped with a bell mouth for a smooth and uniform inflow of air, flow straighteners to straighten the airflow in the wind tunnel, and an adjustable axial fan (Kruger, SG) to provide air suction at 2 m/s.

### Fractal-fin configuration

3D SPTV application on fractal-induced turbulence over a plate-fin array is conducted based on the numerically optimized fractal-fin configuration proposed by Hoi et al.^11,12^. They investigated the optimization of plate-fin heat sink forced convective heat transfer as a function of the 2D planar space-filling square fractal grid (SFG) iteration number *N*, fractal thickness ratio *t*_*r*_, plate-fin heat sink inter-fin distance *δ*, and grid-fin separation *ℓ* in a 0.5334 m × 0.5715 m rectangular wind tunnel using the Computational Fluids Dynamics (CFD) solver, ANSYS-Fluent. Hoi et al.^11^ discovered that, despite having an equivalent blockage ratio, SFG_3_ (*N* = 3) generated thermofluids interplays outperform RFG_2_ (*N* = 2) and SFG_4_ (*N* = 4) by 3.7% and 5.4%, respectively, in predominant forced convection. Their findings demonstrate that the turbulence intensity and velocity generated leeward of RFG_2_ and SFG_4_ are relatively weaker than SFG_3_, leading to insignificant fluid flow restructuring within the fins that facilitates thermal dissipation. As a result, an optimum fractal grid and plate-fin geometrical combination having *N* = 3, *t*_*r*_ = 9.77, *δ* = 0.005 m, and *ℓ* = 0.01 m was proposed. This configuration delivers Nusselt number of 3661.0 which is 6.1% and 16.3% higher than that of the control case and the least favourable configuration, respectively^11,12^. However, it is important to note that the optimized fractal-fin geometrical configuration is secured for flow Reynolds number of *Re*_*Dh*_ = 20.3 × 10^3^ (following the typical HVAC’s flow rate, i.e. 2 ms^-1^). A variation in *Re*_*Dh*_ would significantly influence the fluid flow characteristics and turbulence profile, thereby a careful fine-tuning of fractal-fin geometrical parameters (*N*, *t*_*r*_, *δ* and *ℓ*) allows the realization of maximum plate-fin heat sink heat transfer. The correlations between Reynolds number and fractal-fin configuration merit further investigation, yet the main interest of this study is to acquire a fundamental comprehension of fractal-induced turbulence on the augmentation of fins forced convection. Thus, the flow dynamics induced via Hoi et al.^11,12^ numerically optimized fractal-fin conformation is employed using SPTV detection at *Re*_*Dh*_ = 20.3 × 10^3^, to further unravel the underlying turbulence management on fins thermal dissipation.

**Figure 5:**
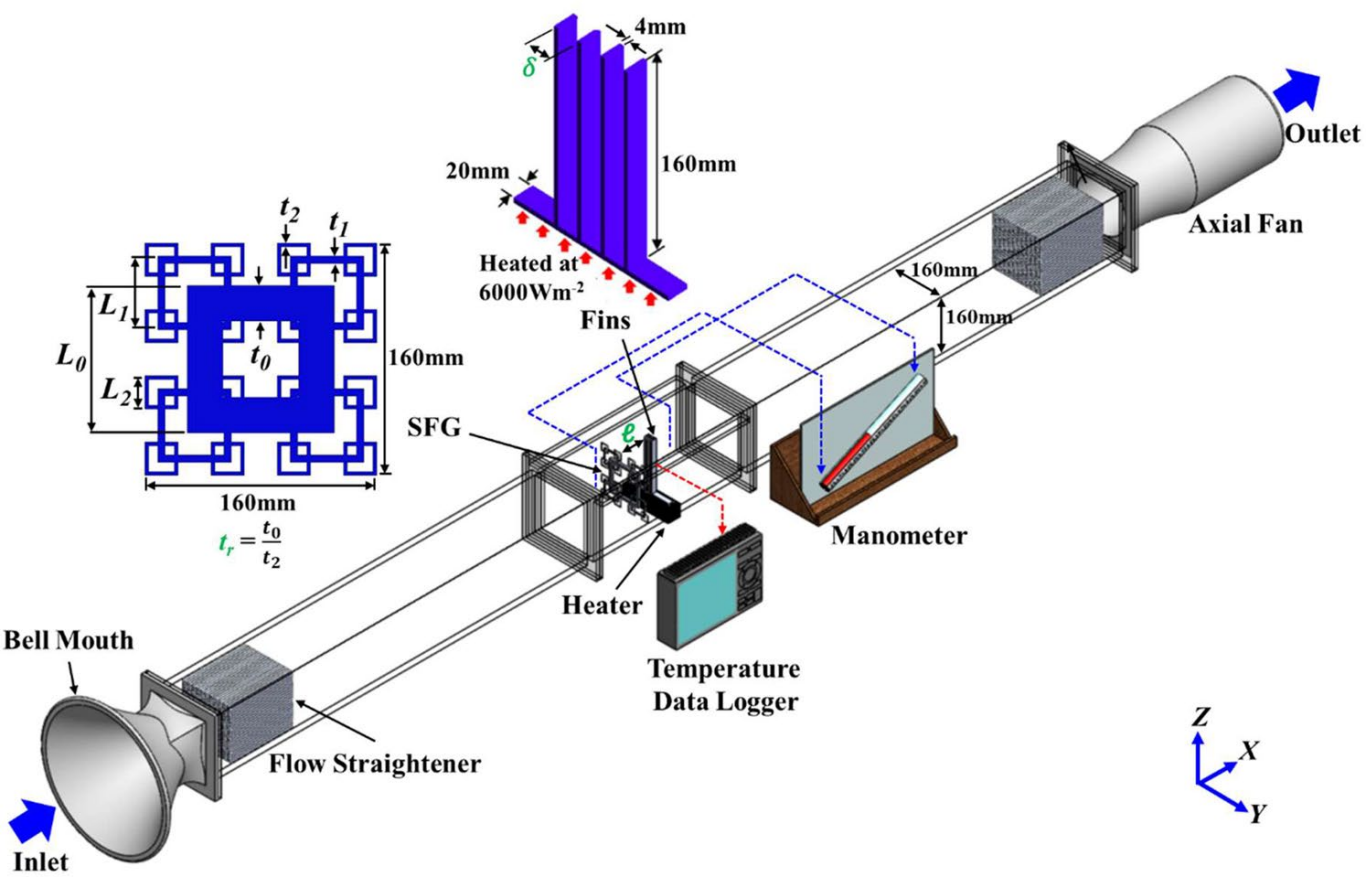
Experimental setup for plate-fin heat sink heat transfer under insert generated turbulent flow.

**Figure 6:**
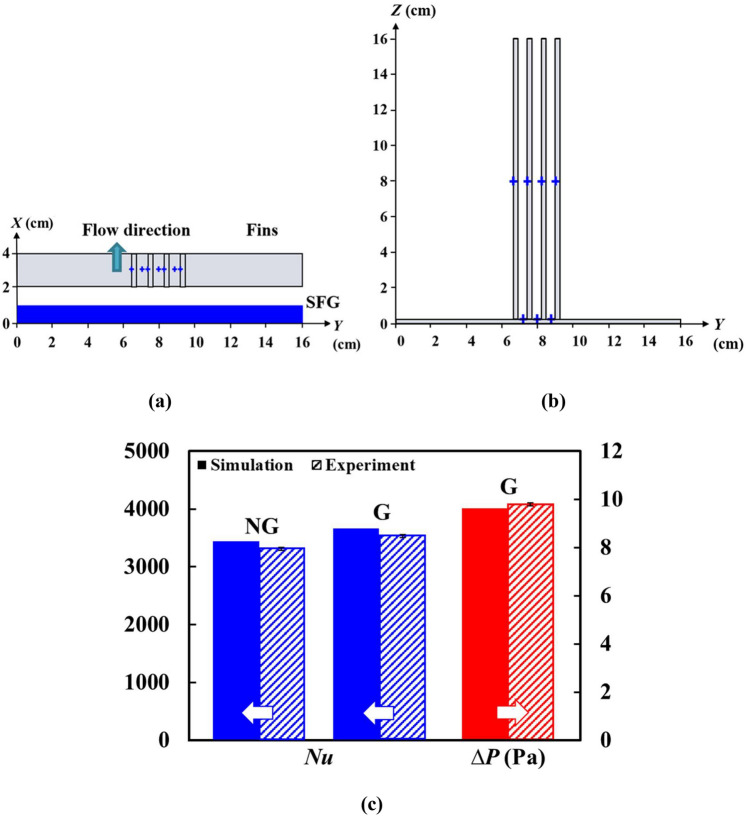
Forced convective heat transfer of Hoi et al.’s^12^ numerically optimized *N* = 3 SFG: the (**a**) top-, (**b**) front-view of the experimentally recoded thermocouple positions on plate-fin heat sink, and (**c**) the experimental verification against Hoi et al.^12^. Note: NG is no grid, and G is with grid.

Prior to SPTV implementation, verification and experimental validation against Hoi et al.’s^12^ numerical study is performed to ascertain the adequacy of the 3D simulation. Figure 5 depicts the experimental setup of the plate-fin heat sink forced convection with insert generated turbulence using Hoi et al.’s^12^ optimized fractal-fin configuration. Sketches of the three iterations SFG and plate-fin heat sink are also provided. The former is made of a 10 mm thick transparent acrylic sheet through laser cutting, and its corresponding geometrical dimensions are provided in Table 1. The latter is manufactured using aluminium 1100-H14 of thermal conductivity 218Wm^−1^ K^−1^ and is constantly heated at 6000 Wm^−2^ by a heater plate (GUNT, DE). A total of seven *T*-type thermocouples are secured onto the heat sink for temperature measurements, and are recorded using Graphtec data logger (GL800, US). The arrangement of the thermocouples on the plate-fin heat sink is illustrated in Fig. 6a, b. The pressure drop across fractal-fin is recorded using an inclined manometer (AIRFLOW Type 5, UK). All data are taken for three minutes with a recording interval of 0.1 s at steady-state condition where the aluminium heat sink has been constantly heated for at least one hour under a constant inflow velocity *U*_*∞*_ of 2 ms^−1^. Moreover, the experiment is repeated 10 times to substantiate the repeatability and stability of the measurements. As demonstrated in Fig. 6c, the experimentally obtained Nusselt number *Nu* for both with and without employing fractal grid, as well as pressure drop ∆*P* for the former are in good agreement with a difference of 4% from Hoi et al.’s^12^ numerical results. This corroborates the accuracy of the numerical findings. Hence, it can be confidently inferred that the profound thermal performance augmentation using CFD-RSO optimized fractal-fin configuration is well-founded, encouraging a deeper insight into the influence of fractal-induced turbulent flow structure on plate-fin heat sink forced convection.

**Table 1:** Dimensions of 2D planar space-filling square fractal grid of *N* = 3.

Parameters	*N* = 3
*L*_0_ (mm)	101.43
*L*_1_ (mm)	50.71
*L*_2_ (mm)	22.86
*t*_0_ (mm)	24.44
*t*_1_ (mm)	5.0
*t*_2_ (mm)	2.5

**Figure 7:**
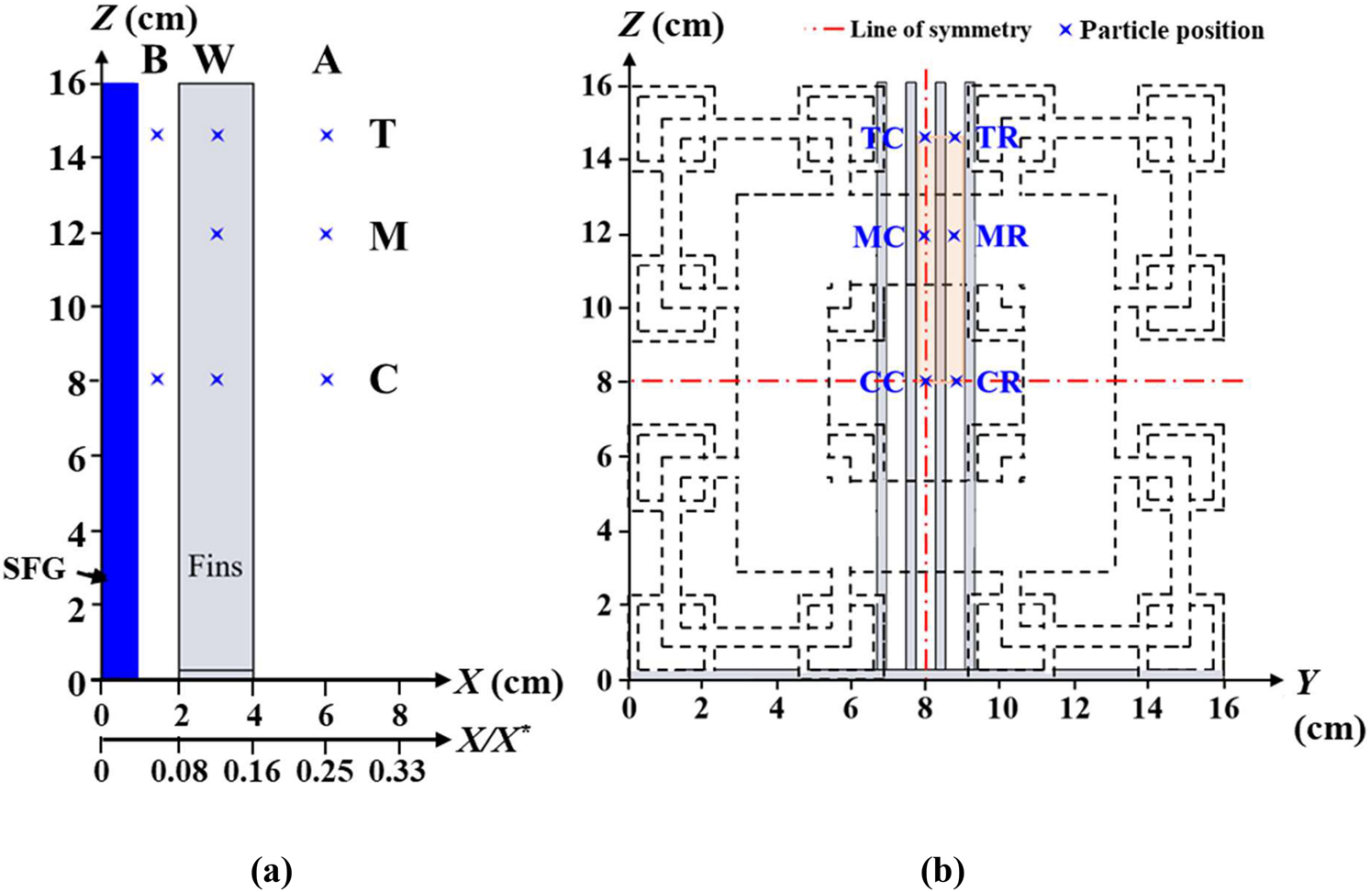
Illustrations of SPTV measured locations (marked by stars) in (**a**) streamwise *X*–*Z* plane and (**b**) spanwise *Y*–*Z* plane. Note: B is position before fins, W is within fins, A is after fins, and (T, M, C, R) indicate (top, middle, centre, right side).

In the current study, the aforementioned 3D SPTV system is employed to explore the hydrodynamics associated with the optimized SFG-fin configuration (G) such that the fluid flow features, which offer 6.1% of *Nu* enhancement, can be explored. In order to allow optical access in between fins, the plate-fin array is made identical to Hoi et al.^12^ using non-conductive transparent acrylic sheets. On this account, forced convection is not considered in the SPTV application as the primary concern of the study is to unveil the effective fractal-induced turbulent flow dynamics on fins that are responsible for heat transfer enhancement. The fractal-fin configuration is positioned at a streamwise location, which is thirteen hydraulic diameters (*D*_*h*_ = 0.16 m) downstream of the inlet to minimize the entrance effects. The origin of the coordinates (*X*, *Y*, *Z*) is positioned at the windward of the fractal grid. Coordinates *X*, *Y* and *Z* are the streamwise, vertical and spanwise directions, respectively. SPTV is applied at regions before, within and after the transparent fins [see Fig. 7]. The SPTV particle is allowed to fluctuate along (i) second iteration grid bar centreline (*Z* = 14.86 cm), which is signified by “T”, (ii) first iteration grid bar centreline (*Z* = 11.85 cm), represented by “M”, and, (iii) fractal grid cross-section centreline (*Z* = 8 cm), denoted as “C”. The flow field is assumed symmetric across *Y* = 0 cm and *Z* = 8 cm; therefore, only the upper-right portion is considered. For each SPTV detection, the tracer particle is followed up over *N*_*F*_ = 4.8 × 10^3^ image-frames, which correspond to a duration of 60 s. Besides, it is important to note that the flow dynamics of fin array without the perturbation of SFG-generated turbulence (NG) is also conducted, such that clear justifications of the favourable turbulence characteristics that promote thermal dissipation can be identified. All the measurements for the control NG and G cases are observed under the Reynolds number of *Re*_*Dh*_ = 20.3 × 10^3^ and are repeated seven times to ensure experimental repeatability.

## Results and discussion

### Equivalent turbulence intensity *I*_*T*,*E*_

**Figure 8:**
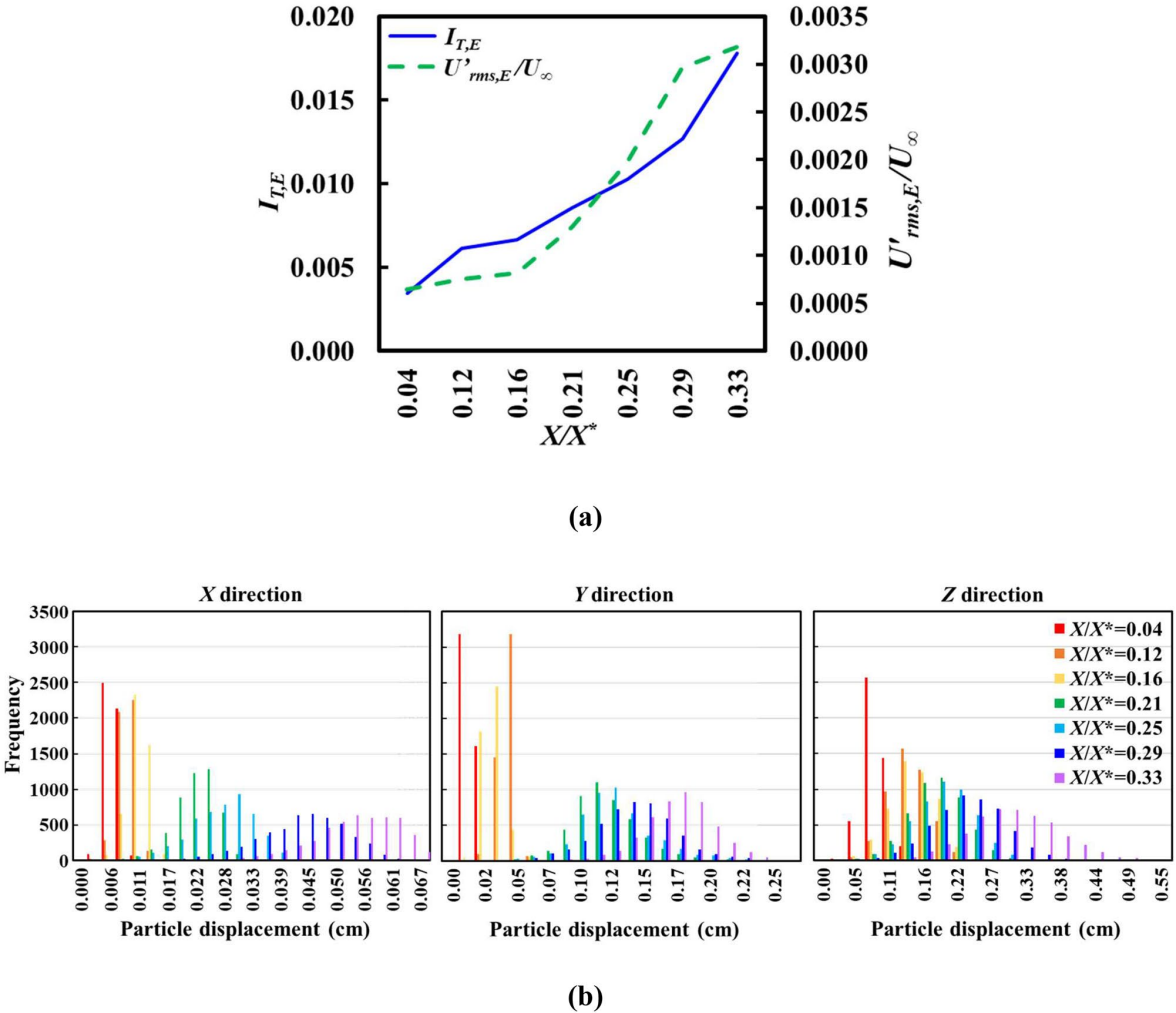
SFG generated centreline (**a**) *I*_*T,E*_ and *U’*_*rms,E*_/*U*_*∞*_, and (**b**) the particle displacement histogram in *X*, *Y*, and *Z* direction at various distances leeward of fractal grid obtained via SPTV.

Turbulence intensity is a crucial indicator in expressing a randomized fluid flow fluctuating strength of high Reynolds number flow dynamics. It is the ratio of the root-mean-square turbulent velocity fluctuations $U_{rms}^{^{\prime}}$ to the mean velocity *U*_*∞*_, namely,

3 $$I_{T,E} = \frac{{U_{rms}^{^{\prime}} }}{{U_{\infty } }}$$

where $U_{rms}^{^{\prime}} = \sqrt {\frac{1}{3}\left( {u^{^{\prime}2} + v^{^{\prime}2} + w^{^{\prime}2} } \right)}$, with *u’*, *v’* and *w’* denoting the velocity fluctuations along the *X*, *Y* and *Z* axes*,* respectively. Subscript “*E*” (Equivalent) represents the regional fluid flow characteristics, following the basic nature of the present SPTV system. To date, the prevailing notion among researchers is that forced convective heat transfer coefficient increases with turbulence intensity via a substantial restructuring of flow-boundary layer fundamental characteristics^12,35,37,41,74^. Melina et al.^37^ and Paul et al.^38^ reported that the production region of SFG-induced turbulence has a stronger dominance over heat transfer than the decay region, despite the fact that the turbulence intensity in both regimes was kept similar. Figure 8a demonstrates the profile of SFG-induced centreline *I*_*T,E*_ and *U’*_*rms,E*_/*U*_*∞*_ (without the presence of fins) along the streamline direction, X/X^*^ where *X* is normalised using the wake interaction length scale as defined by Mazellier and Vassilicos^42^, viz. X^*^ = $L_{0}^{2} /t_{0}$. Undoubtedly, the present control volume (X/X* < 0.33) leeward of SFG is situated within the turbulence production region, i.e., the turbulence intensity rises with distance downstream X/X*. Interestingly, it is observed that the tracer particle fluctuates more vigorously with greater particle displacement coverage in *X*, *Y* and *Z* directions as the flow travels further downstream [see Fig. 8b]. This is mainly due to the strong interactions of vortices and wakes originated from the multilength-scale fractal grid bars, hence providing the energy necessary for turbulence production. As elucidated by Mazellier and Vassilicos^42^, the wakes emanating leeward of SFG is strictly a function of fractal grid dimension and downstream distance from the grid, where larger bars create bigger wakes that meet and interact further downstream from grid^42^. As a consequence, *I*_*T,E*_ and *U’*_*rms,E*_/*U*_*∞*_ increase with X/X* in the production region, as larger wakes contribute to stronger and more violent wake interplays.

**Figure 9:**
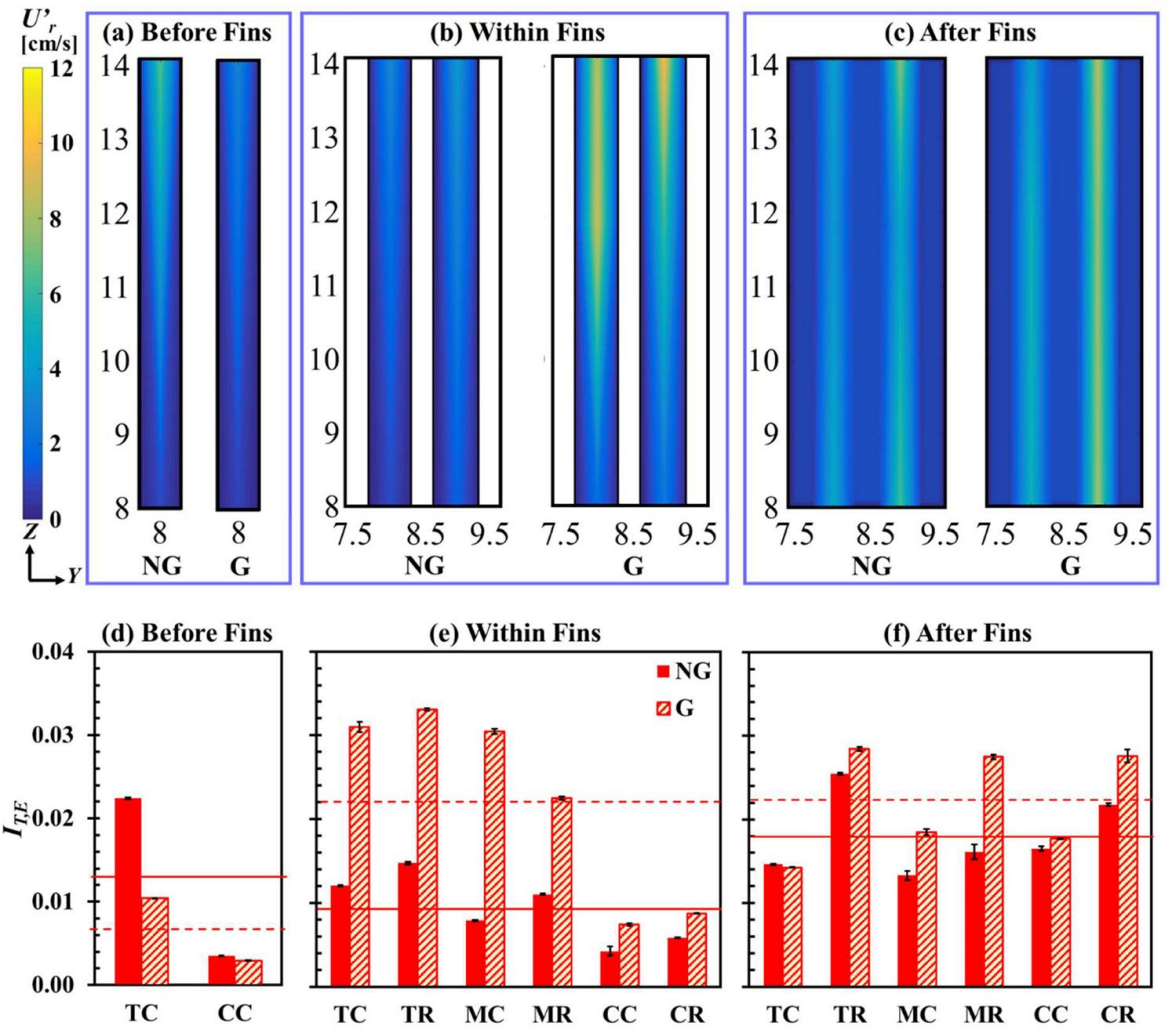
2D contours of SPTV detected particle velocity fluctuation *U*’_*r*_ on *Y*–*Z* plane (top row) and SPTV measured equivalent turbulence intensity *I*_*T,E*_ (bottom row) for NG and G at positions (**a**, **d**) before fins, (**b**, **e**) within fins and (**c**, **f**) after fins. Note: the horizontal solid line indicates average value for NG whereas dashed line represents average value for G.

The SFG generated resultant velocity fluctuation *U*’_*r*_ and equivalent turbulence intensity, *I*_*T,E*_ before (X/X* = 0.06), within (X/X* = 0.12) and after (X/X* = 0.25) the fin array, in the aforementioned optimized fractal-fin configuration, is illustrated in Fig. 9. The 2D contours of *U*’_*r*_ demonstrate that velocity fluctuations before fins are greater for NG than G [see Fig. 9a]. In the presence of SFG, flow separation and low-velocity flow recirculation formed immediately leeward of the bars of SFG, leading to weaker flow fluctuations within the region between SFG and fins for G as compared to control NG. Inversely, the flow velocity fluctuations are stronger for G than NG at positions within fins and after fins [see Fig. 9b, c]. The solid plate-fin array imposes physical disruption to the flow recirculation downstream of the SFG, thereby significantly altering the velocity profile within the fins. Clearly, the optimized fractal-fin configuration is a mutually complementary combination that is able to effectively restructure the fluid flow structure and boundary layer around the fins, heightening the flow velocity fluctuations within the fins. As shown in Fig. 9c, the flow fluctuations downstream of the fin array varies from those within fins, owing to the formation of flow separations and vortices at each fin’s trailing edge.

Remarkably, the equivalent turbulence intensity, *I*_*T,E*_ before fins for NG is higher than G, especially at the location TC [see Fig. 9d]. It is clear that as position TC is relatively closer to the channel wall, the effect of fluid flow perturbation is rather apparent. A higher near-wall velocity gradient with *Re*_*Dh*_ of 20.3 × 10^3^ yields greater wall shear stress, inducing sturdy turbulence eddies. Unequivocally, it is observed that the presence of SFG allows the restructuring of flow dynamics, resulting in a lower *I*_*T,E*_ than that of the control NG, immediately leeward of SFG. As aforementioned, fractal grids induce multilength-scales turbulence with smaller wakes interacting closer to the grid and larger wakes interacting further from the grid. Thus, the turbulence intensity immediately leeward of the fractal grid and before fins is low, due to the weak small scale wakes interactions. Additionally, the small scale wakes close to the grid may not interact effectively with the near-wall turbulence eddies, leading to a lower *I*_*T,E*_ for G as compared to NG at position TC before fins. Consequently, the averaged *I*_*T,E*_ before fins for G is lower than NG. On another note, the wall turbulence impact reduces considerably while moving towards the centre of the wind tunnel at CC, of which, the *I*_*T,E*_ for both cases is lower.

Once the air reaches the fin array, the flow characteristics significantly varied. As seen in Fig. 9e, the *I*_*T,E*_ for G is higher than NG, regardless of tracer particle locations, with an averaged *I*_*T,E*_ augmentation of 138.7%. SFG imposes channel obstruction, which accelerates the airflow to effectively penetrate through the sudden grid contraction into the fin array downstream. As a consequence, the accelerated airflow through the fin array gives rise to higher wall-shear stress in the fin boundary layer, leading to a thinner viscous sub-layer for G than NG^75^. Moreover, the interplay between local flow accelerations and wakes generated leeward of individual grid bar generates a pronounced amount of shear that stimulates multilength-scale turbulent eddies formation. The turbulent eddies of smaller length scales may potentially be filtered into the fin array to integrate and further agitate the flow structures within the fin-surface flow-boundary layer. Lastly, the existence of solid fins physically disrupts the wakes of larger length-scale that shed from SFG. Hence, the forced through airflow unique incorporation of the former primitive lower length-scale SFG-induced turbulence, as well as the latter interference of larger length-scale eddies with fins, eventually lead to an unconventional flow dynamics of higher turbulence intensity to predominantly reshuffle the fin-surface flow-boundary layers, and most importantly, promote plate-fin heat sink forced convection.

The fluid flow fluctuations at X/X* = 0.25 in the lee of fins are shown in Fig. 9f. Clearly, the averaged *I*_*T,E*_ for G remains high while the *I*_*T,E*_ for NG further increases. The undulating pressure downwind of fins incites vortices shedding from the heat sink trailing edge. The interactions between these vortices contribute significantly to the escalation of NG’s *I*_*T,E*_. In addition, the larger wakes originating from SFG’s thicker grid bars, which has yet to be cascaded further, may intervene with each of the fin trailing edge induced wakes and vortices, hence arousing a heighten in *I*_*T,E*_ for G than NG. Moreover, owing to the wide spectrum of anisotropic and inhomogeneous fluid flow fluctuations that a 2D planar space-filling square fractal grid produces, such variety of *I*_*T,E*_ for G is found to incur a total of 36.8% enhancement than that of the control NG.

### Equivalent integral length scale *L*_*i*,*E*_

**Figure 10:**
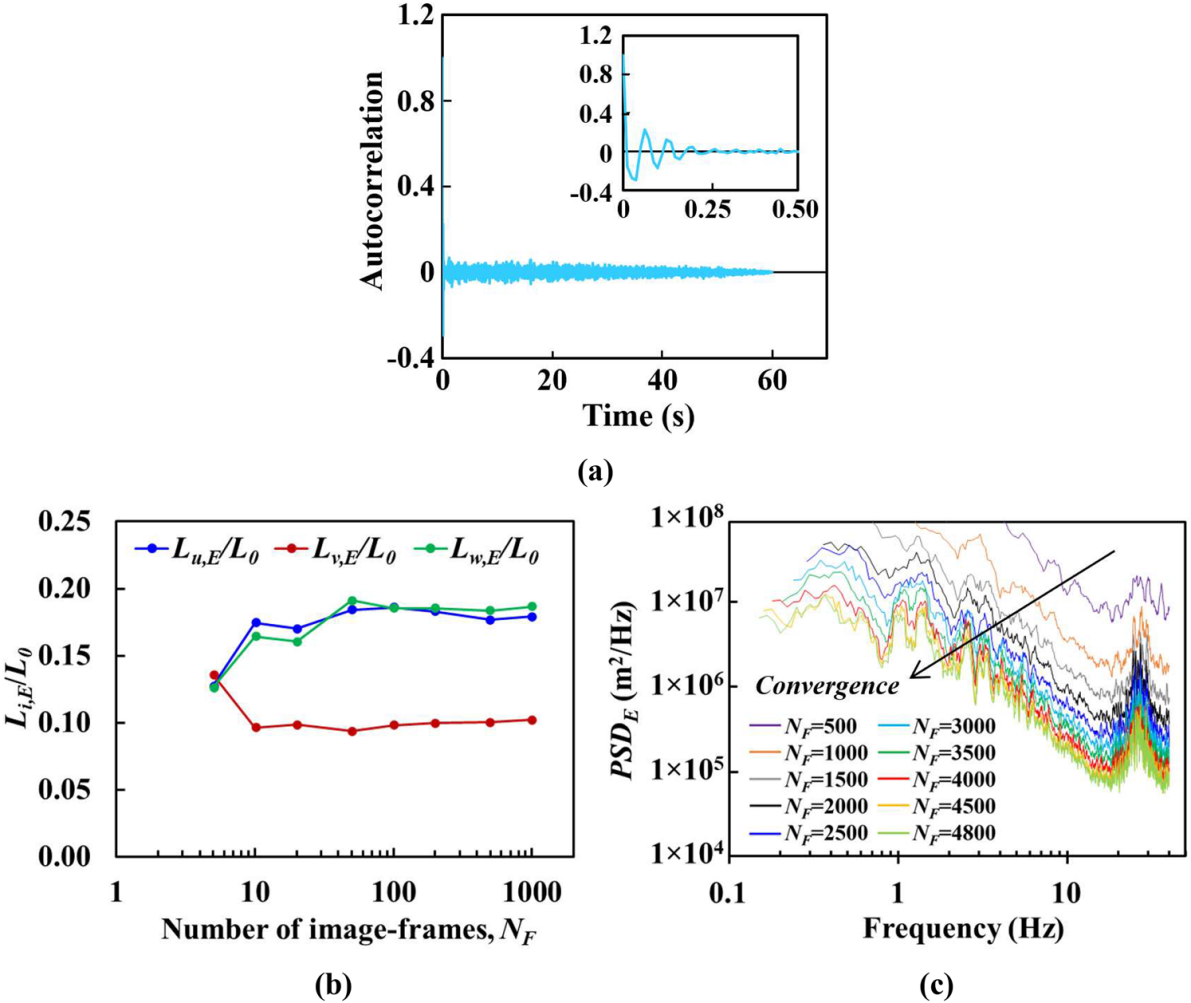
Illustration of (**a**) autocorrelation function of velocity fluctuations, convergence study of (**b**) equivalent integral length scale *L*_*i,E*_*/L*_*0*_ and (**c**) equivalent power spectral density *PSD*_*E*_ with increasing number of image-frames, *N*_*F*_—Case G at position TC within fins.

Turbulence length scale indicates a spatial dimension of eddies (or vortices) deduced from velocity fluctuations of high Reynolds number fluid flow. The large-scale eddies within such intricate multilength-scale flow structures play crucial roles in transporting fluid flow momentum, as well as contorting the interface between the wakes and the irrotational fluid before being cascaded down to inertial subrange eddies, which are then further dissipated as heat once reaches towards the smallest Kolmogorov microscale. Hence, this study mainly focuses on the large-scale eddies, namely, of the order of integral length-scale, or the alleged energy-containing eddy length-scale, in unravelling the fractal grid induced flow dynamics while flow penetrating through the plate-fin array. Briefly, the present equivalent integral length scale can be secured by means of integrating the autocorrelated fluctuating velocity component along the time-lapse *∆*, up to the first zero-crossing at *T*, namely,

4 $$L_{i,E} = U_{\infty } \mathop \smallint \limits_{0}^{T} R_{{i^{\prime}}} \left( t \right)d\Delta$$

where $R_{{i^{\prime}}} \left( t \right)$ represents the autocorrelation function defined as,

5 $$R_{{i^{\prime } }} \left( t \right) = \frac{{\left\langle {i^{\prime } \left( t \right)i^{\prime } \left( {t + \Delta } \right)} \right\rangle }}{{\left\langle {i^{\prime } \left( t \right)^{2} } \right\rangle }}$$

with <  > denoting the ensemble average and *i'* denoting the fluctuating velocity component *u’*, *v’* and w’^39,76,77^. Figure 10a, b illustrates the autocorrelation function of velocity fluctuations and the convergence of integral length scales with increasing particle tracking time frame for position TC within fins of case G. It is seen that with a sufficient amount of tracking duration, integral length scales can be extracted precisely using Eqs. (4) and (5). The integral length scale convergence for all other positions of both NG and G cases are achieved at image-frames, *N*_*F*_ ≤ 1.5 × 10^3^ with less than 5% about the expected value. In the present study, the integral length scale is normalized using SFG first iterative bar length *L*_*0*_, i.e., *L*_*i,E*_*/L*_*0*_*.* The current order of *L*_*i,E*_*/L*_*0*_ well-matches with Hurst and Vassilicos^21^, as well as Nagata et al.^78^ recorded integral length scale ratios which are in the range of (0.1 to 0.2) and (0.03 to 0.25), respectively.

**Figure 11:**
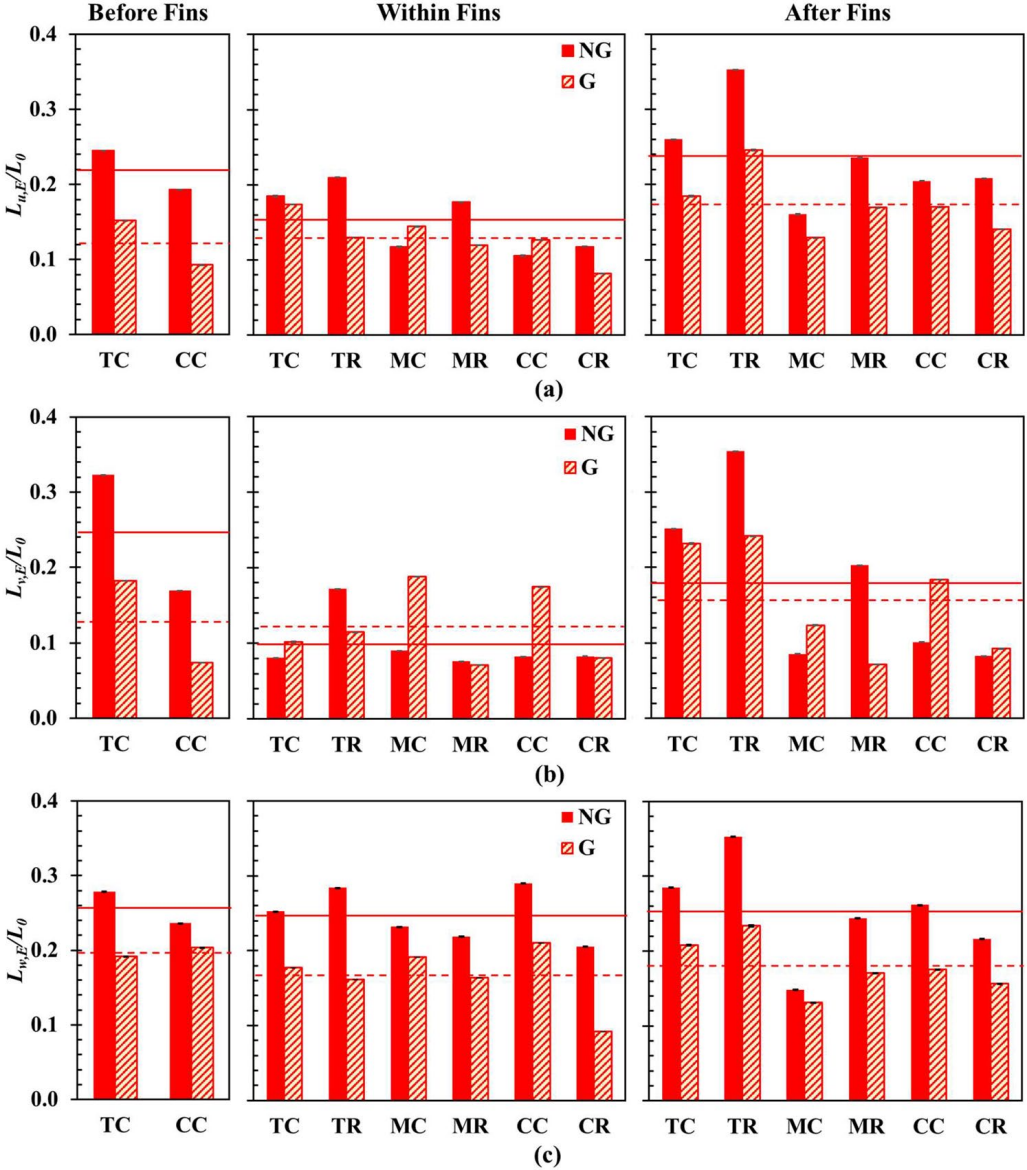
SPTV detected equivalent integral length scale, non-dimensionalized with *L*_*0*_ of the (**a**) streamwise *u*, (**b**) spanwise *v*, and (**c**) cross-stream *w* fluctuating velocity components.

Figure 11 illustrates the SPTV detected *L*_*i,E*_*/L*_*0*_ for longitudinal (*u’*), lateral (*v’*) and transverse (*w’*) flow dynamics. Notably, the SFG-induced turbulent flow is highly inhomogeneous with significant *L*_*i,E*_*/L*_0_ variations in *X*, *Y* and *Z* directions. The overall *L*_*i,E*_*/L*_*0*_ in all the three components for G (dashed line) is mostly smaller than NG (solid line) in upstream, within and downstream of the fin array. This suggests that a smaller integral length scale is desired to improve the heat transfer coefficient. Similar phenomenon was reported by Melina et al.^37^ and Torri and Yang^39^, where they discovered that thermal dissipation increases with decreasing turbulence length scale. In the production region, interaction and mutual straining between the vortices shed from the SFG and fins are inevitable. The vortices either break down into smaller ones or merge into a larger common structure depending on the direction of the vortex rotation. Counter-rotating vortex pairs develop strains that cause shrinkage in vortex diameter, i.e., vortex stretching. The reduction in vortex size increases its angular velocity *ω* as angular momentum is directly proportional to *ωr*^2^ and is conserved. Consequently, the vorticity associated with small eddies is intensified, and thus small-scale eddies comprise greater vorticity than large-scale eddies. It is suggested that the underlying vorticity in small-scale eddies may play a vital role in enhancing heat transfer^79–83^. Interestingly, the averaged lateral *L*_*v,E*_*/L*_*0*_ between fins is larger for G than it would be without the grid. Such phenomenon may indicate that the relatively larger vortices in the transverse direction within fins are able to interrupt the fin flow-boundary layer more effectively. Understandably, fluid flow within fin array for NG and G has a smaller averaged *L*_*v,E*_*/L*_*0*_, as compared to the flow before and after fins as the fin walls set a physical constraint to the size of grid induced eddies. In essence, the fractal-fin configuration is able to give rise to an overall favourable small-scale turbulence that augments heat transfer performance.

### Equivalent skewness and kurtosis of SPTV detected velocity fluctuations

Skewness, *S* and kurtosis, *K* of flow perturbations are evaluated to statistically analyze the basis behaviour of fractal grid induced turbulence. Skewness measures the symmetricity in a distribution in the third central moment of velocity fluctuations, normalized by the variance,

6 $$S_{i,E} = \frac{{\left\langle {i^{^{\prime}3} } \right\rangle }}{{\left\langle {i^{^{\prime}2} } \right\rangle^{3/2} }}$$

Kurtosis, also known as flatness, indicates the tails of a distribution, i.e., a measure of outliers present in a distribution. It is defined as the fourth central moment of velocity fluctuations divided by variance,

7 $$K_{i,E} = \frac{{\left\langle {i^{^{\prime}4} } \right\rangle }}{{\left\langle {i^{^{\prime}2} } \right\rangle^{2} }}$$

**Figure 12:**
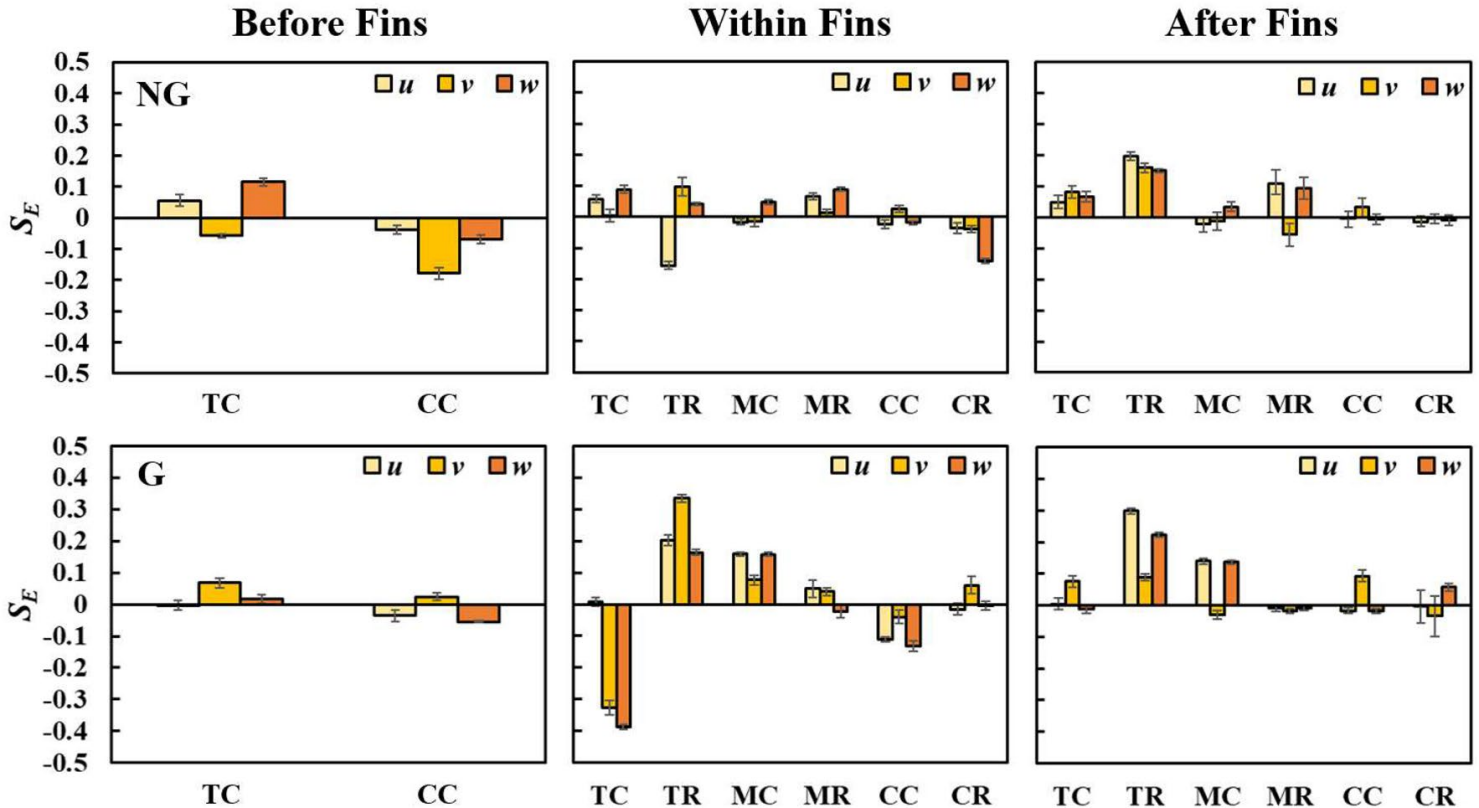
Equivalent skewness of velocity fluctuations along streamwise *u*, spanwise *v* and cross-stream *w* for NG (top row) and G (bottom row).

**Figure 13:**
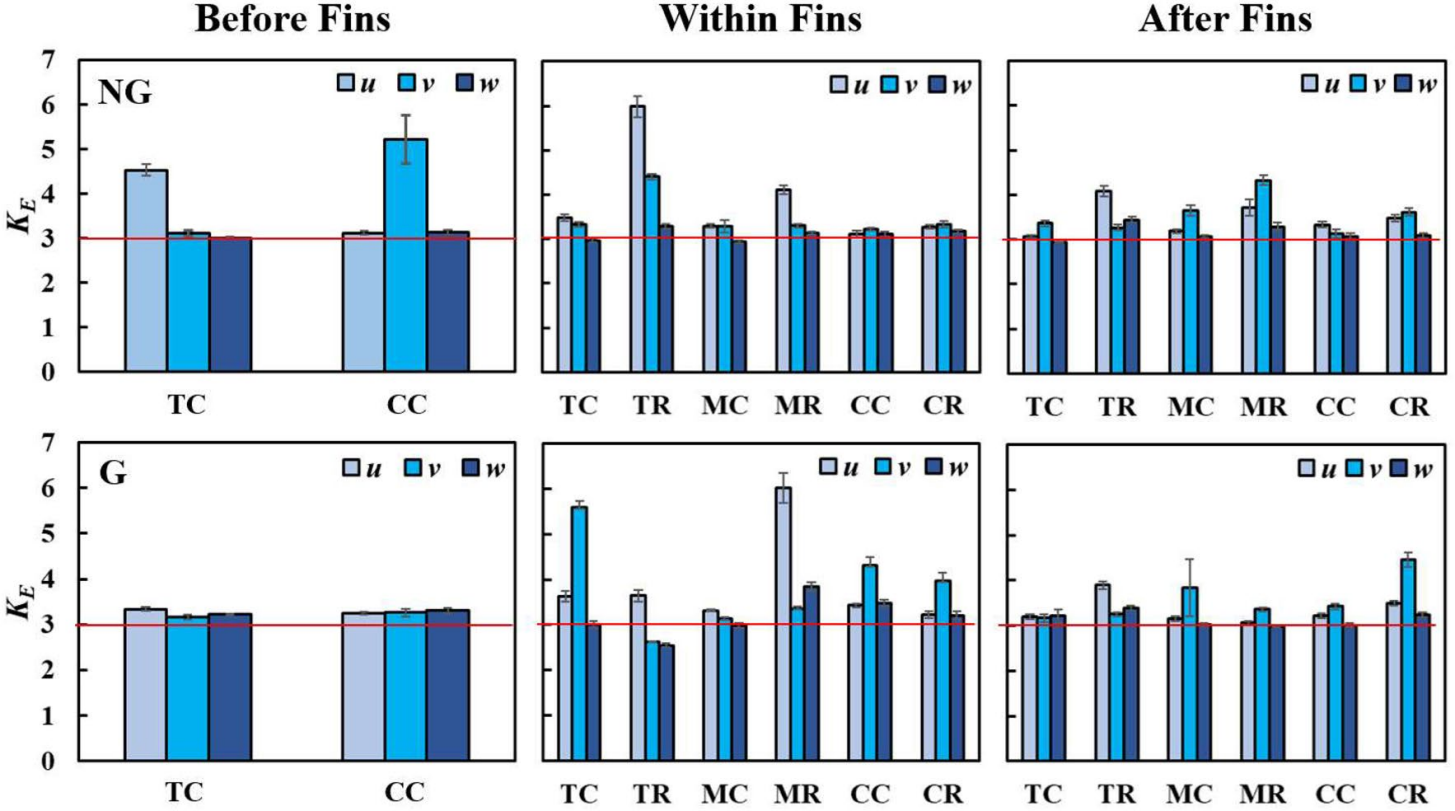
Equivalent kurtosis of velocity fluctuations along streamwise *u*, spanwise *v* and cross-stream *w* for NG (top row) and G (bottom row).

Previous researchers^24,38,42,78,84^ have extensively observed that immediately leeward of fractal insert (*X*/X* < 0.1), the channel centreline velocity fluctuations are Gaussian with skewness of zero and flatness of 3. As the flow travels further downstream (0.1 < *X*/X* < 0.26), the velocity fluctuations appear to be strongly non-Gaussian, with the largest negative values of skewness and the largest positive values of flatness occurring at *X*/X*≈0.2. Later, the skewness and flatness of velocity fluctuations approach Gaussian behaviour at *X*/X* > 0.26. In this study, the equivalent skewness and flatness of *u’*, *v’* and *w’* at various locations are depicted in Fig. 12 and Fig. 13, respectively. At the upstream of fins for NG, the velocity fluctuations deviate slightly from Gaussian behaviour (*S*_*E*_ > 0, *K*_*E*_ > 3). Yet, with the addition of SFG, the velocity fluctuations are highly Gaussian (*S*_*E*_≈0, *K*_*E*_≈3) before fins, i.e., immediately leeward of SFG (*X*/X* = 0.06). Besides, the fractal induced velocity fluctuations at several locations within (*X*/X* = 0.12) and after (*X*/X* = 0.25) the fin array are found to be strongly non-Gaussian. These two observations are consonant with the earlier studies, where velocity fluctuations are Gaussian at *X*/X* < 0.1 but highly non-Gaussian between 0.1 < *X*/X* < 0.26^24,38,42,78,84^. Remarkably, the gaussianity behaviour leeward of SFG (0.12 < *X*/X* < 0.25) is highly local position-dependent. The velocity fluctuations at TC and TR within fins exhibit significant negative and positive *S*_*E*_, respectively. This highlights that the fluid flow at TC experiences local deceleration, while extreme accelerating flow occurs at TR. Besides, *K*_*E*_ is unusually high within fin at TR for NG, and at TC and MR for G. Clearly, the *S*_*E*_ and *K*_*E*_ within fin array vary considerably with location due to the inclusion of SFG. In particular, higher *I*_*T,E*_, *L*_*v,E*_/*L*_*0*_, *S*_*E*_(*v*, *w*) and *K*_*E*_(*v*) in magnitude, which gives rise to additional complex flow dynamic interplays, are observed at position TC and CC for G, as compared to NG. Hence, it can be inferred that highly skewed velocity fluctuation and intense rare turbulent events may be favourable in the potent restructuring of the flow-boundary layer within fin array, leading towards betterment in forced convective thermal dissipation.

### Equivalent Power spectral density *PSD*_*E*_

**Figure 14:**
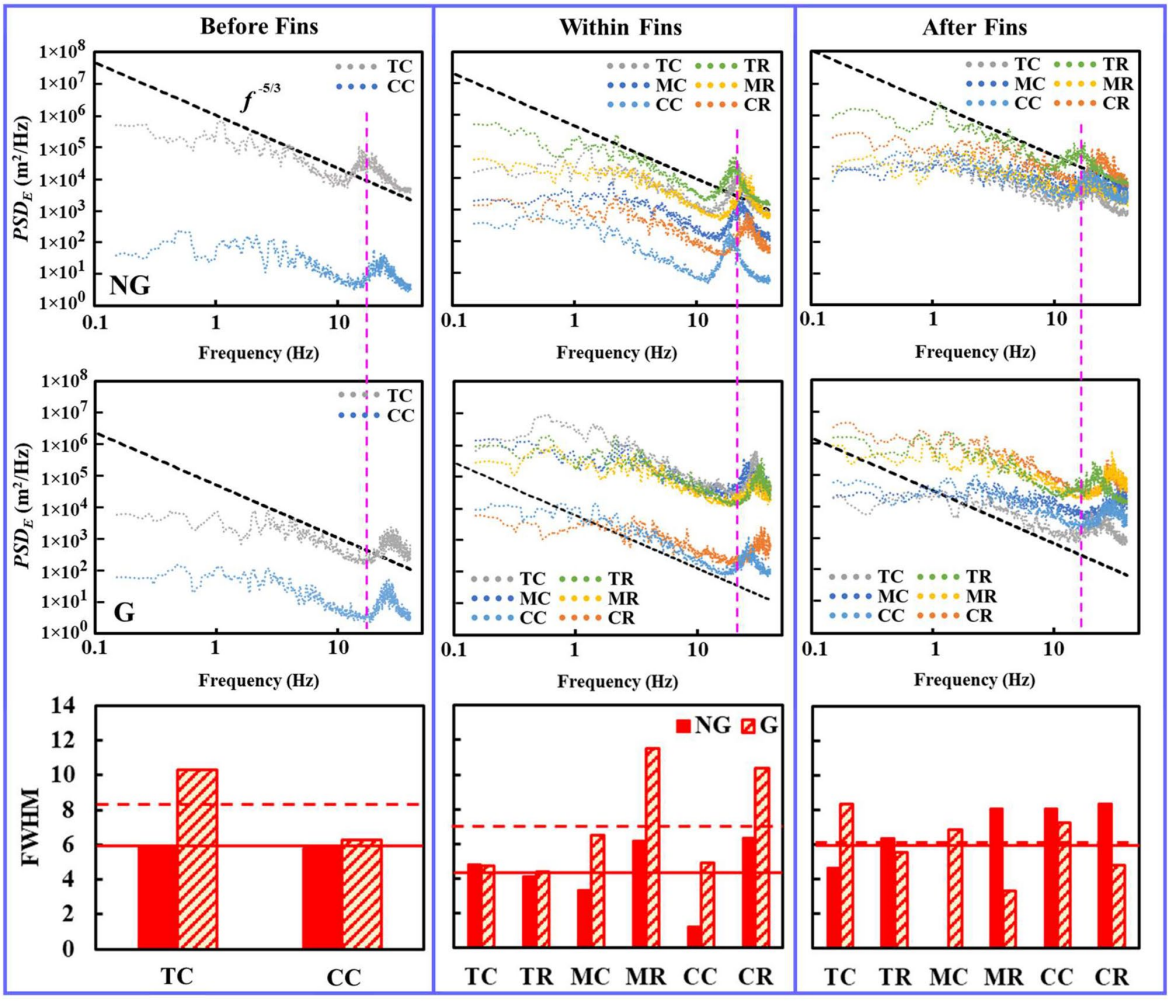
The 10-moving averaged equivalent *PSD*_*E*_ (m^2^/Hz) of velocity fluctuations at *Re*_*Dh*_ of 20.3 × 10^3^: top row: NG, middle: G, and bottom: the corresponding full width at half maximum of the peaks (FWHM). Vertical dashed reference lines illustrate the shift of spectra peaks between NG and G.

The stochastic SFG-induced turbulence can be further deciphered into frequency power spectra, namely, the power spectral density (*PSD*), to disclose the strength of velocity fluctuations in the frequency domain. Hence, *PSD* is determined by taking the Fourier transformations of the autocovariance function with respect to fluid flow undulating time (*t*) histories [*u’*(*t*), *w’*(*t*), *v’*(*t*)], defined as,

8 $$S\left( f \right) = \mathop \smallint \limits_{ - \infty }^{ + \infty } \rho_{i} \left( t \right)e^{ - j2\pi ft} dt$$

9 $$\rho_{i} \left( t \right) = \left\langle {i^{\prime } \left( t \right)i^{\prime } \left( {t + \Delta } \right)} \right\rangle$$

where *S*(*f*) represents the *PSD*, $\rho_{{i^{\prime}}} \left( t \right)$ the autocovariance function of velocity fluctuations, and *j* the imaginary unit^37^. Convergence of *PSD*_*E*_ with an increasing number of image-frames is depicted in Fig. 10c for case G at position TC within fins. This effectively demonstrates that the tracking duration of 60 s with image-frames number *N*_*F*_ = 4.8 × 10^3^ is appropriate for *PSD*_*E*_ convergence within 7% from the expected value. The equivalent power spectra density *PSD*_*E*_ for the velocity fluctuations measured at *Re*_*Dh*_ = 20.3 × 10^3^ via SPTV are presented in Fig. 14. A 10-lapse period moving average is applied to the *PSD*_*E*_ to lessen the random noises. Unambiguously, the frequency *f* is inversely proportional to the size of the turbulence eddies, i.e., a large energy-containing eddy is accompanying with low-*f*. As the frequency increases, energy decreases; most importantly, the reduction of energy exhibits the Kolmogorov’s classical decay law with exponents approaching −5/3 over a broad range of *f* for both NG and G, despite the fact that the measurements for G are in the heart of turbulence production regime. This puzzling phenomenon was also revealed by Melina et al.^84^, Laziet and Vassilicos^22^ and Fernandes et al.^85^_,_ who looked into fractal-induced turbulence characteristics, particularly in the turbulence production region. At high-frequency sub-range, spectra peaks are observed with frequency of peak magnitude varying with the measuring positions as well as with the implementation of SFG. Such fundamental velocity fluctuations are attributed to coherent structure flow dynamics, with wake vortex shedding giving rise to the strong peaks in the power spectra^22,37,86,87^. The vortex shedding event appears over a broad range of frequency and energy, which indicates that vortex shedding involves multilength-scale eddies of different energy levels. It is interesting to note that the full width at half maximum (FWHM) of the maximum energy amplitude is wider for G at locations before and within fins. In this regard, SFG’s bars induce vortex shedding that gives broader scales of high energy eddy than NG. Besides, the vortex shedding events occurred at a higher frequency with the addition of SFG, regardless of the position of the measurements [see vertical dashed lines in Fig. 14]. Last but not least, it is seen that *PSD*_*E*_ levels for G within and after fins are higher than NG. This phenomenon is in line with the behaviour of *I*_*T,E*_ as discussed in Sect. 3.1, which implies that *PSD*_*E*_ is highly correlated with the turbulence intensity, i.e., locations with high turbulence intensity give high *PSD*_*E*_. This is consistent with previously reported results in the literature^84,88^. More specifically, Melina et al.^84^ suggested that more intense vortex shedding contributes to higher turbulence intensity. In short, at X/X* = 0.12 (TC and CC), higher *I*_*T,E*_, *L*_*v,E*_/*L*_*0*_, |*S*_*E*_(*v*, *w*)| and *K*_*E*_(*v*) together with the higher spreading of *PSD*_*E*_ are determined for G, which may shed light, and more importantly, to support the fact that energetic small-scale vortex shedding at high frequency are preferred for the flow dynamics of fractal-induced turbulence over a plate-fin array forced convection.

## Conclusion

A 3D stationary particle tracking velocimetry (SPTV) is uniquely developed with the aims to provide a low-cost off-the-shelf solution to the HVAC industry, and most importantly, to shed more light on the influence of 2D planar space-filling square fractal grid induced turbulence in promoting the forced convective heat transfer of a validated numerically optimized grid-fin configuration. The main outcomes of the present study are,

1. The maximum percentage differences of the present 3D SPTV have manifested the positional precisions of 0.43%, 0.51% and 0.78%, which correspond to the absolute deviations of approximately 4.3 × 10^–3^ cm, 5.1 × 10^-3^ cm and 7.8 × 10^-3^ cm, respectively, in the (*X*, *Y*, *Z*) coordinates as compared to the actual predefined distances. Hence, assuring the current novel recursive corrective algorithm in acquiring and attaining high positional accuracy, reliability and repeatability.
2. The instantaneous regional fluid flow characteristics have been successfully realized using the current SPTV. It is capable to elucidate the nature of chaotic fractal-induced turbulence (particularly within a 1 × 4 fin array), namely, high turbulence intensity attributed to multilength-scale wake interactions, Kolmogorov’s −5/3 law decay, vortex shedding events, and Gaussian behaviour immediately leeward of the *N* = 3 SFG followed by non-Gaussian features between 0.12 < *X*/X* < 0.25.
3. By comparing between the control NG and G in the regions before, within and after a fin array, SPTV reveals the fact that vigorous turbulence intensity, small integral length scale and energetic high-frequency vortex shedding are dynamically critical and preferred for the generation of hydrodynamic that could ultimately give rise to a substantial heat transfer enhancement of fractal-induced plate-fin thermal dissipation.
4. For region within the confined fin spacing at *X*/X* = 0.12, higher *I*_*T,E*_, *L*_*v,E*_/*L*_*0*_, |*S*_*E*_(*v*, *w*)|, *K*_*E*_(*v*) and *PSD*_*E*_ are determined for G at position TC and CC than those of the control NG. Hence, demonstrating the possible fluid flow structures and mechanisms induced to predominately reshuffle the flow-boundary layer along fin-surface in augmenting forced convection.
5. The essence of SPTV resides in its simplicity of application, low cost worldwide available materials, and most importantly, it is non-intrusive and is applicable in a confined workspace, i.e., the precious thermal effective flow dynamics detection within the inter-fin spacings *δ* of a plate-fin heat sink. Unequivocally, such flow dynamics detection shall be able to better facilitate the development, or perhaps to reformulate the commercially available HVAC finned-tube heat exchanger configuration of *δ* < 2 mm.
6. Naturally, employing an inexpensive SPTV system in place of costly, high-end professional flow measurement methods is inevitably accompanied by some trade-offs. A 1 mm EPS particle is employed in place of a commercially available expensive airflow tracing particle. Owing to the particle size of 1 mm, the current SPTV system measurement is limited to length scales that are larger than 1 mm.
7. This SPTV study is conducted in the absence of plate-fin heat transfer to provide optical access within the fins. In fact, with the presence of heat transfer, the flow structure and the strength of turbulence may vary due to the additional buoyancy force exerted by temperature-induced fluid-property variations.

## Data Availability

Data are available from the authors upon reasonable request and with the permission of Monash University.

## Abbreviations

*A*_*d*_: Channel cross-sectional area, cm^2^
*D*_*h*_: Hydraulic diameter, 4*A*_*d*_/*P*_*d*_, m
*d*_*p*_: Particle diameter, mm
*I*_*T,E*_: Equivalent turbulence intensity
*K*_*i,E*_: Equivalent flatness of velocity fluctuations
*L*_*0*_: Length of thickest SFG bar, m
*L*_*i,E*_: Equivalent integral length scale, m
*L*_*x*_: Duct length, m
*L*_*y*_: Duct width, m
*L*_*z*_: Duct height, m
*N*: Number of fractal iteration
*N*_*F*_: Number of image-frames
*Nu*: Nusselt number
Δ*P*: Pressure drop, Pa
*P*_*d*_: Duct perimeter, m
*PSD*_*E*_: Equivalent power spectra density
*r*: Radius of vortex, m
*Re*_*Dh*_: Hydraulic diameter based Reynolds number, *ρ**U*_*∞*_*D*_*h*_*/μ*
SFG: 2D planar space-filling square fractal grid
*S*_*i*__,__*E*_: Equivalent skewness of velocity fluctuations
*S*_*t*_: Stokes number
*t*_*r*_: Thickness ratio
*u**'*: Velocity fluctuation, cm/s
*U’*_*r*_: Resultant velocity fluctuation, cm/s
*U*_*∞*_: Velocity inlet, m/s
*X**: Wake interaction length scale

## Greek symbols

*δ*: Inter-fin distance, m
*ℓ*: Grid-fin distance, m
*μ*: Dynamic viscosity of air, kg/ms
*ν*: Kinematic viscosity of air, m^2^/s
*ρ*: Density of air, kg/m^3^
*ρ*_*p*_: Density of particle, kg/m^3^
*ω*: Vortex angular momentum, rad/s
*τ*_*p*_: Particle response time, s
*τ*_*f*_: Characteristic timescale of fluid flow, s
